# Peptide chain release factor DIG8 regulates plant growth by affecting ROS-mediated sugar transportation in *Arabidopsis thaliana*

**DOI:** 10.3389/fpls.2023.1172275

**Published:** 2023-03-31

**Authors:** Xiangxiang Zhang, Yuliang Han, Xiao Han, Siqi Zhang, Liming Xiong, Tao Chen

**Affiliations:** ^1^ Zhejiang Provincial Key Laboratory for Genetic Improvement and Quality Control of Medicinal Plants, College of Life and Environmental Science, Hangzhou Normal University, Hangzhou, China; ^2^ College of Life Sciences, Fuzhou University, Fuzhou, China; ^3^ Department of Biology, Hong Kong Baptist University, Kowloon Tang, Hong Kong, Hong Kong SAR, China

**Keywords:** chloroplast, peptide chain release factor, ROS, callose, sugar transportation

## Abstract

Chloroplasts have important roles in photosynthesis, stress sensing and retrograde signaling. However, the relationship between chloroplast peptide chain release factor and ROS-mediated plant growth is still unclear. In the present study, we obtained a loss-of-function mutant *dig8* by EMS mutation. The *dig8* mutant has few lateral roots and a pale green leaf phenotype. By map-based cloning, the *DIG8* gene was located on AT3G62910, with a point mutation leading to amino acid substitution in functional release factor domain. Using yeast-two-hybrid and BiFC, we confirmed *DIG8* protein was characterized locating in chloroplast by co-localization with plastid marker and interacting with ribosome-related proteins. Through observing by transmission electron microscopy, quantifying ROS content and measuring the transport efficiency of plasmodesmata in dig8 mutant, we found that abnormal thylakoid stack formation and chloroplast dysfunction in the *dig8* mutant caused increased ROS activity leading to callose deposition and lower PD permeability. A local sugar supplement partially alleviated the growth retardation phenotype of the mutant. These findings shed light on chloroplast peptide chain release factor-affected plant growth by ROS stress.

## 1 Introduction

Photosynthesis in plant chloroplasts, the most important organelles in higher plants, converts light energy to chemical energy and fixes carbon, which are stored into sugar. Chloroplasts are differentiated plastids evolved from a cyanobacterial ancestor by endosymbiosis in a eukaryotic host cell (Raven and Allen, 2003; Dyall et al., 2004). The chloroplast genome in higher plants encodes about 100 genes, but the overwhelming majority of chloroplast-located proteins are encoded by nuclear genes (Abdallah et al., 2000).

A chloroplast genome contains about 40 photosynthesis-related and 60 housekeeping genes that are essential for protein synthesis (Sato et al., 1999). To date, protein synthesis events in chloroplast have been intensively explored, while the mechanism of translation termination remaining largely unknown (Zoschke and Bock, 2018). Three types of peptide chain release factors have been characterized in *Escherichia coli* (Scolnick et al., 1968; Craigen and Caskey, 1986; Gao et al., 2007; Zaher and Green, 2011) and two types, prfA and prfB, have been characterized in Arabidopsis plastids (Meurer et al., 1998; Meurer et al., 2002; Stoppel et al., 2011). prfA and prfB are responsible for UAA/UAG and UAA/UGA, respectively, and are recognized by the first and third amino acids in the highly conserved motifs in P-A(V/Q)-T of prfA and S-P-F of prfB (Scolnick et al., 1968; Nakamura and Ito, 1998; Ito et al., 2000; Nakamura et al., 2000; Nakamura and Ito, 2002). The GGQ motif in both prfA and prfB is essential for peptidyl-tRNA hydrolysis (Frolova et al., 1999; Dinçbas-Renqvist et al., 2000; Song et al., 2000; Vestergaard et al., 2001; Mora et al., 2003). Peptide chain release factors have two functions in translation termination, viz. recognition of the stop codon and catalysis of peptidyl-tRNA hydrolysis. Mutations in peptide chain release factor genes cause abnormal phenotypes exampled by dysfunction of chloroplasts in Arabidopsis (Meurer et al., 2002; Stoppel et al., 2011; Nick et al., 2013).

The chloroplast is an important site for reactive oxygen species (ROS) production (Foyer and Noctor, 2005; Asada, 2006). ROS are reactive forms of molecular oxygen, including singlet oxygen (^1^O_2_), superoxide radicals ( ${\text{O}}_{2}^{-}$ ), hydrogen peroxide (H_2_O_2_) and hydroxyl radicals (OH.). The photosynthetic electron transport chain (PET) in chloroplasts is a constant source of ROS, and these ROS cause oxidative stress when at excessive levels (Woodson, 2016). ROS plays important roles in transduction of intracellular signals and in control of gene expression and activity of antioxidant systems (Desikan et al., 2001; Apel and Hirt, 2004; Mullineaux et al., 2006; Foyer and Shigeoka, 2011). Exposure to stress in plants can lead to a sudden drastic increase in ROS production which was called an “oxidative burst” (Desikan et al., 2001). Generation and accumulation of ROS caused by imbalanced metabolic activities that are very sensitive to environmental stimuli lead to changes in ion fluxes and gene expression, oxidation of cellular components, disrupted organelle integrity and consequent effects on plant developmental processes such as embryogenesis, leaf growth, root branching, stoma opening and closing, and flowering. Therefore, ROS production must be fine-tuned to maintain redox homeostasis (Suzuki et al., 2012).

Here we report on the characterization of the *dig8* (drought inhibited growth of lateral roots) mutant originally isolated by its abnormal lateral root development (Xiong et al., 2006). In addition to fewer lateral roots, the mutant has pale green leaves, dwarf stature and other abnormalities. The *DIG8* gene was identified by map-based mapping and cloning and found to encode a chloroplast-localized peptide chain release factor with vital roles in chloroplast development and function. Dysfunction molecular transportation in the *dig8* mutant caused abnormal chloroplast development causing higher levels of ROS activity leading to plant growth retardation. We provide evidence linking the chloroplast development and ROS-mediated callose deposition, and propose that the normal chloroplasts are essential for transportation through plasmodesmata in *Arabidopsis*.

## 2 Result

### 2.1 Isolation and characterization of the *dig8* mutant

**Figure 1:**
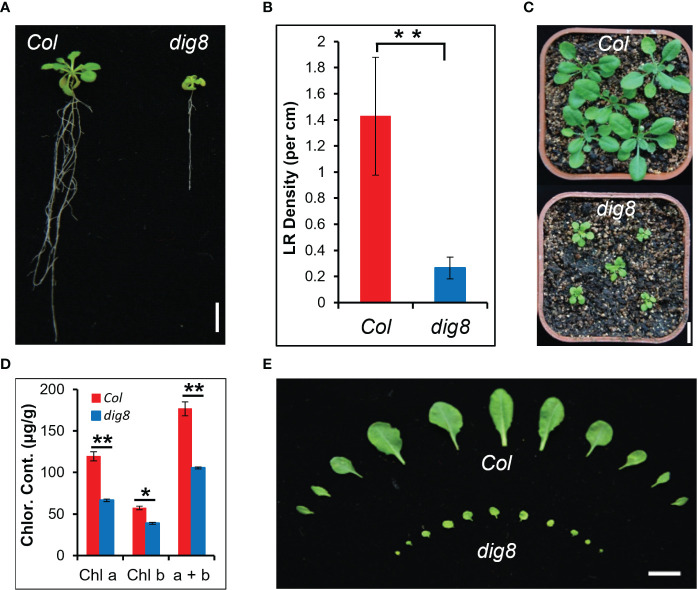
Identification of the *dig8* mutant. **(A)**, The *dig8* mutant has fewer lateral roots. Scale bar, 1 cm. **(B)**, Lateral root (LR) density in the *dig8* mutant and Col. Error bars represent SD (n=30). **P < 0.01 (Student’s *t* test). **(C)**, Morphology of Col and *dig8* seedlings in soil. Scale bar, 1 cm. **(D)**, Chlorophyll contents in Col and *dig8* mutant. Shown are means of chlorophyll a, chlorophyll b and their total contents. Error bars represent SD from three replicates. *P < 0.05 and **P < 0.01 (Student’s *t* test). **(E)**, Comparison of the rosette leaves at transition from the vegetative to the reproductive stage. Scale Bar, 1 cm.

Osmotic stress is an influential environmental stimulus that can inhibit lateral root growth in Arabidopsis. This effect was used to establish a screening system for mutants affecting osmotic stress response. Mutant *dig8* was isolated from an M_2_ generation of an EMS-mutagenized population in the Columbia (Col) background by selecting for defective lateral root development. As shown in **Figure 1A**, the *dig8* mutant had a shorter primary root and fewer lateral roots compared to the wildtype (WT). The lateral root density of the *dig8* mutant was one-seventh that of Col (**Figure 1B**). Seedlings of the *dig8* mutant had smaller leaves and shorter petioles with the same rosette number (**Figures 1C, E**). Reduced chlorophyll contents in the *dig8* mutant (**Figure 1D**) caused pale green cotyledons and leaves (**Figure S1**).

**Figure 2:**
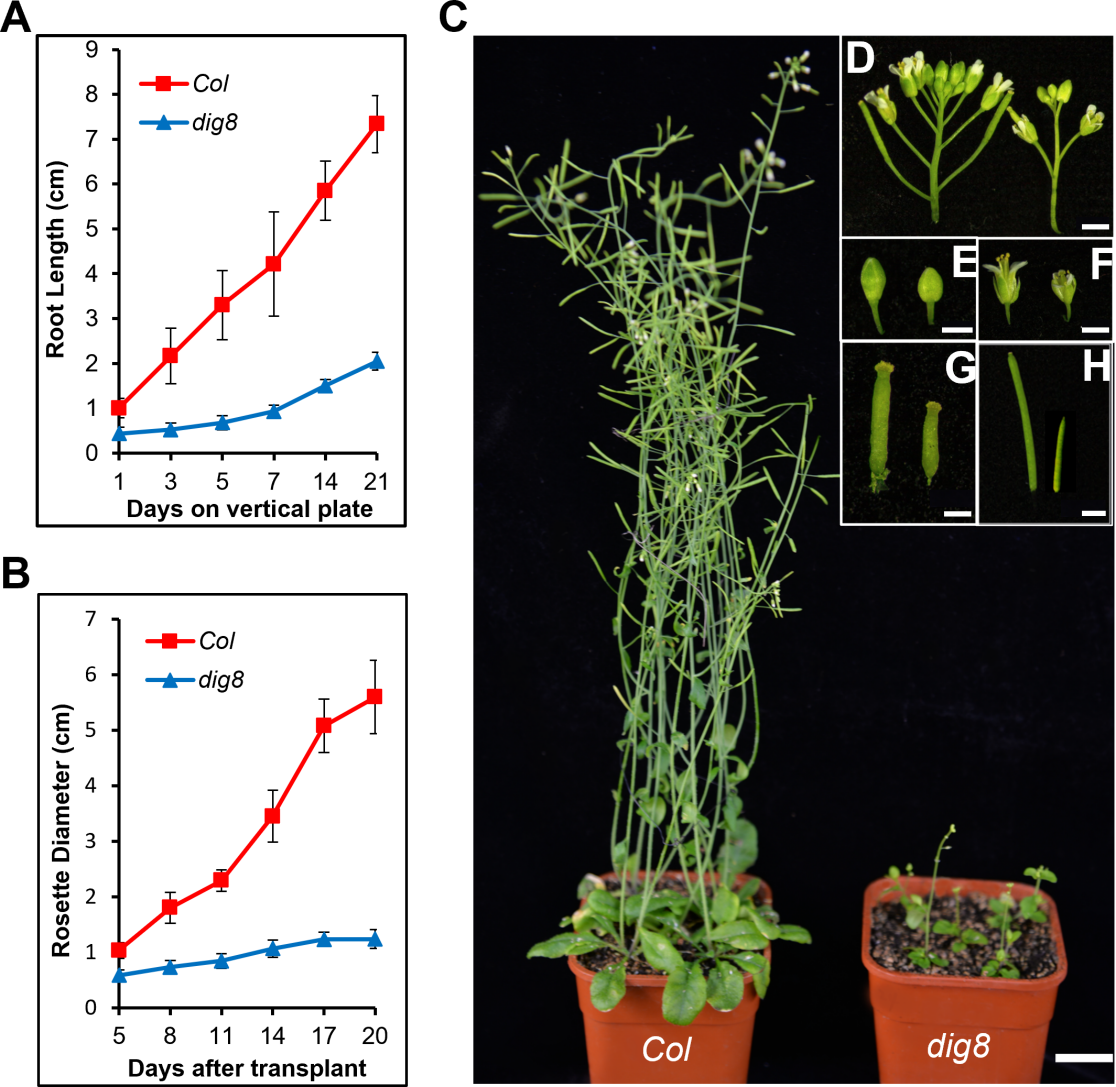
Retarded growth of the *dig8* mutant. **(A)**, Root growth rates of Col and *dig8* mutant on a vertical plate. 10-day-old seedlings were transferred to a vertically placed plate and root length was recorded daily for 3 weeks. Error bars represent SD (n=20). **(B)**, Growth rate measured daily by rosette diameters. Error bars represent SD (n=20). **(C)**, Phenotypes of Col and *dig8* mutant adult plants. Scale bar, 2 cm. **(D–H)**, Enlarged view of flowers and siliques of Col (on the left) and *dig8* mutant (on the right). **(D)**, inflorescences; **(E)**, flower buds; **(F)**, pollinated flowers; **(G)**, developing siliques; **(H)**, siliques. Scale bars, 0.2 cm.

To compare growth traits, we transferred 10-day-old WT and mutant seedlings to the vertical plate and made daily measurements of root growth. The root growth rate of the *dig8* mutant was much slower than that of Col (**Figure 2A**). Following transplanting to soil in pots the growth rate of the mutant was also much slower than that of the WT (**Figure 2B**). Overall the mutant showed extreme dwarfness, but the flowering timing was similar to that of Col (**Figure 2C**); the mutant had a sparse inflorescence with few small flower buds (**Figures 2D, E**), tiny petals, short styles, and short stamens (**Figures 2F, G**). The siliques of *dig8* mutant were shorter than those of Col but the seeds were significantly larger (**Figures 2H**, **S2**).

### 2.2 *DIG8* encodes a chloroplast protein release factor

**Figure 3:**
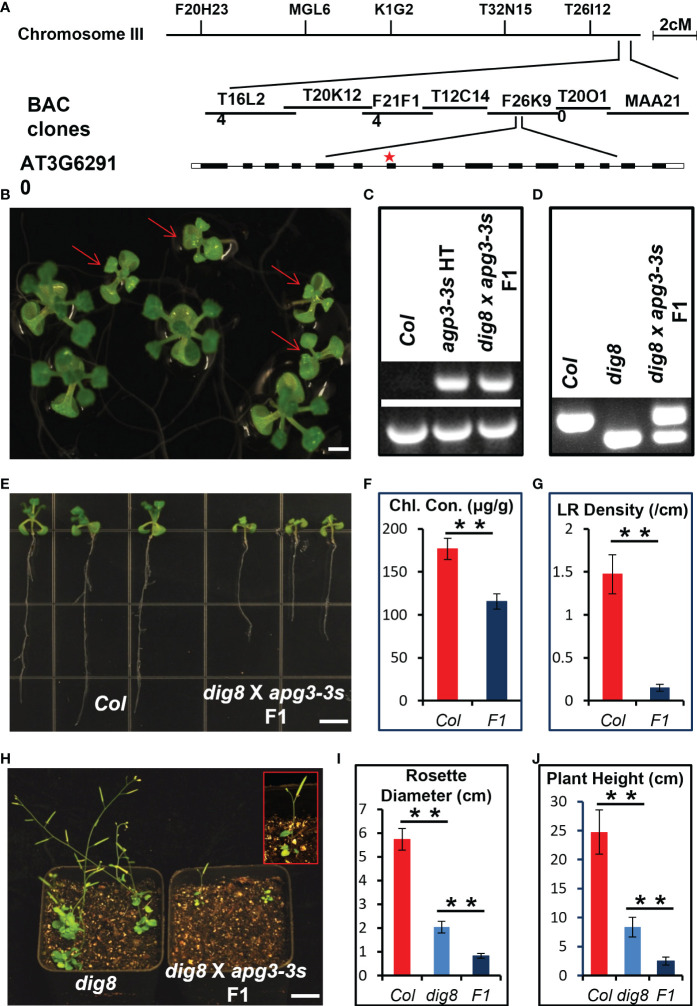
Mapping and allelic verification of *DIG8*. **(A)**, Map-based cloning of the *DIG8* locus. The *dig8* mutant was crossed with the Ler ecotype and the F_2_ population was used for mapping. *DIG8* was localized on Chromosome 3. Schematic structure of the *DIG8* gene (AT3G62910); black boxes, exons; white boxes, UTRs; lines, introns; star, *dig8* mutation site. **(B)**, Allelic verification of *DIG8* gene. Then *dig8* mutant was crossed with an *apg3-3s* heterozygote. F_1_ seedlings on MS medium showed segregation ratio of 1 normal: 1 defective (arrows) seedling as expected for allelic mutations. Scale bars, 0.2 cm. **(C)**, PCR verification of T-DNA insertion in the *apg3-3s* mutant allele. Lower bands were generated by PCR using primers LP and RP; upper bands by PCR using primers LB and RP. **(D)**, dCAPS verification of *dig8* mutant site. The BglII site was designed in reverse primer for *dig8* mutant site. PCR products were digested with BglII to verify the point mutation in *dig8* mutant. **(E)**, Lateral root and pale green phenotypes of *dig8* x *apg3-3s* F_1_ seedlings compared to Col. **(F)**, Total chlorophyll contents in Col and *dig8* x *apg3-3s* F_1_ seedlings. Error bars represent SD from three independent replicates. **(G)**, Lateral root (LR) densities of Col and F_1_ mutant seedlings. Error bars represent SD (n=20). Scale bars, 0.5 cm. **(H)**, Comparison of *dig8* x *apg3-3s* F_1_ with *dig8* mutant. Enlarged view of a *dig8* x *apg3-3s* F_1_ plant in red rectangle showing 3 rosette leaves and 1 silique at the reproductive stage. Scale bars, 1 cm. I and J, Comparisons of rosette width **(I)** and plant height **(J)** of Col, *dig8* and *dig8* x *apg3-3s* F_1_ seedlings. Error bars represent SD (*n*=20). Asterisks indicate a significant difference between the indicated samples (*t*-test, **P < 0.01).

To map the *DIG8* locus, a mapping population was generated by crossing the *dig8* mutant with the ecotype Landsberg *erecta* (Ler). The mutation was first mapped to the end of Chromosome 3 between BAC clones T16L24 and MAA21. Fine mapping located *DIG8* gene in clone F26K9. Sequencing identified a single G-to-A nucleotide change in the gene AT3G62910. This mutation was predicted to cause a glycine to arginine conversion at amino acid residue 147 in the translated peptide (**Figures 3A**, **S3**). This gene encodes a peptide chain release factor and a mutant in this gene was earlier named *APG3* (albino or pale green mutant 3) based the pale green phenotype (Motohashi et al., 2007). The three-dimensional structure of the protein predicted by Phyre2 showed that the basic structure was made up of alpha-helices (**Figure S4A**). As seen in **Figure S4B** the conserved PCRF (peptide chain release factor) domain of the DIG8 protein is located at 119-234 a.a. and the mutant 147 site is highly conserved relative to other surrounding residues. This indicated that the mutant protein could have weakened or partial loss-of-function in translation termination. Alignment with the peptide chain release factors in both nuclei and chloroplasts revealed that DIG8 was a chloroplast-located protein (**Figure S5A**). With a conserved GGQ motif and P-A(V/Q)-T motif, DIG8 was considered to be a prfA protein (**Figure S5B**).

Genetic allelism test was performed to determine whether DIG8 and APG3 were alleles. We crossed the *dig8* mutant with an *apg3-3s* (T-DNA insertion mutant SAIL_1251_H05), heterozygote since the *apg3-3s* homozygous was lethal (**Figure S6**). The phenotypes of *dig8* mutant x *apg3-3s* heterozygote F_1_ seedlings segregated 1 normal: 1 mutant (**Figure 3B**). We confirmed that the *dig8* x *apg3-3s* F_1_ plants with mutant phenotype were heterozygous for the T-DNA insertion and heterozygous for *dig8* point mutation, revealing that *DIG8* and *APG3* were alleles (**Figures 3C, D**). As shown in **Figures 3E–G**, the mutant F_1_ seedlings had pale green cotyledons and leaves, and a shorter primary root and hardly any lateral roots compared with Col. Interestingly, these plants had only 3 rosette leaves, 1 silique and were approximately 2 cm in height. These phenotypes were much more severe than those found in the *dig8* mutant (**Figures 3H–J**).

### 2.3 Expression pattern and functional interaction of *DIG8*

**Figure 4:**
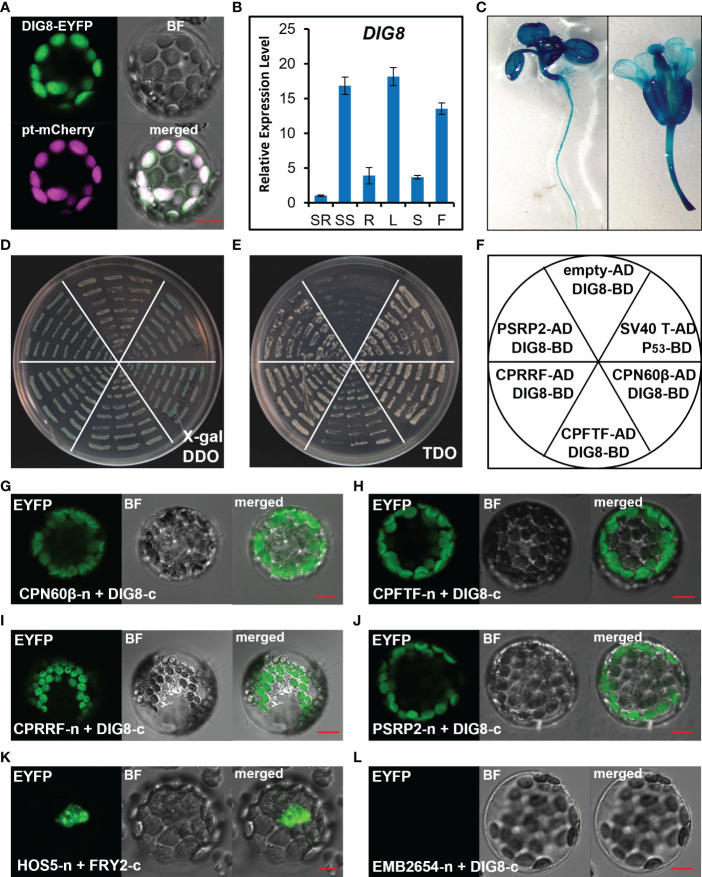
Characterization of the DIG8 protein. **(A)**, DIG8 co-localized with a plastid marker. A DIG8-EYFP construct was co-transformed with plastid-localized marker pt-mCherry in protoplasts. The fluorescence signals were detected by confocal microscopy. BF, bright field. **(B)**, qRT-PCR analysis of *DIG8* expression. SR, seedling root; SS, seedling shoot; R, root; L, leaf; S, stem; F, flower. *ACT2* was used as the internal control in qRT-PCR assays. Error bars represent the standard deviations (n=3). **(C)**, *DIG8* promoter-driven GUS expression. Transgenic plants expressing GUS under control of the *DIG8* promoter were stained with X-Gluc and imaged under a microscope. **(D–F)**, Confirmation of interactions between DIG8 and predicted DIG8- interacting proteins in the yeast 2-hybrid system. DIG8 was subcloned into the BD vector. CPN60β, CPFTF, CPRRF, PSRP2 were subcloned into the AD vector. DIG8-BD was co-transformed into yeast with each AD construct. The interactions were expressed on DDO medium with X-gal **(D)** and TDO medium **(E)**. **(F)**, Arrangement of yeast strains shown in **(D)** and **(E)**. The empty-AD and DIG8-BD pair was used as a negative control, and the SV40T-AD and P53-BD pair was used as a positive control. **(G–L)**, Confirmation of interactions between DIG8 and predicted DIG8-interacting proteins in protoplasts. DIG8 was subcloned into the c-EYFP vector; the others were subcloned into the n-EYFP vector. DIG8-c was co-transformed into protoplasts with each n-YFP construct. The EYFP signals of DIG8-c with CPN60β-n **(G)**, CPFTF-n **(H)**, CPRRF-n **(I)**, PSRP2-n **(J)** were observed by confocal microscopy. **(K)**, Interaction of HOS5-n and FRY2-c was used as the positive control. **(L)**, Interaction of EMB2654-n and DIG8-c was used the negative control. Scale bars=10 µm.

Since DIG8 was predicted to be a peptide release factor in chloroplasts, we generated a DIG8-GFP fusion construct and co-transferred it with a chloroplast localization marker into chloroplasts. As shown in **Figure 4A**, DIG8 co-localized with the marker in chloroplasts. To reveal the temporal-spatial expression pattern of *DIG8*, we performed qRT-PCR and promoter-GUS analysis. *DIG8* was dominantly expressed in shoot tissue at the seedling stage, in leaves and flowers at the reproductive stage (**Figures 4B, C**).

To test the possible function of DIG8 in plastid protein synthesis regulation, we carried out yeast-two-hybrid assays to verify for interaction of DIG8 with ribosome components and a new peptide folding chaperonin. The β subunit of the chloroplast chaperonin 60 (CPN60β, AT1G55490), chloroplast protein folding trigger factor (CPFTF, AT2G30695), plastid-specific ribosomal protein 2 (PSRP2, AT3G52150), and chloroplast ribosome recycling factor (CPRRF, AT3G63190) were selected to confirm interaction with DIG8. Co-transformed yeast clones were transferred to the DDO medium with X-gal and TDO medium along with positive and negative controls. All selected yeast clones turned blue in the presence of X-gal and grew on the TDO medium, indicating that DIG8 interacted with these chloroplast ribosome-related proteins (**Figures 4D–F**). To confirm the locations of interactions in chloroplasts, we generated BiFC constructs in which DIG8 was fused with C-YFP and other proteins were fused with N-YFP. Signals for interaction in chloroplasts were detected by transient expression in protoplasts. Consistent with earlier results in yeast, DIG8 showed strong interaction with CPN60β, CPFTF, PSRP2 and CPRRF in plant cells (**Figures 4G–L**).

### 2.4 Thylakoid stack disequilibrium and chloroplast dysfunction in the *dig8* mutant

**Figure 5:**
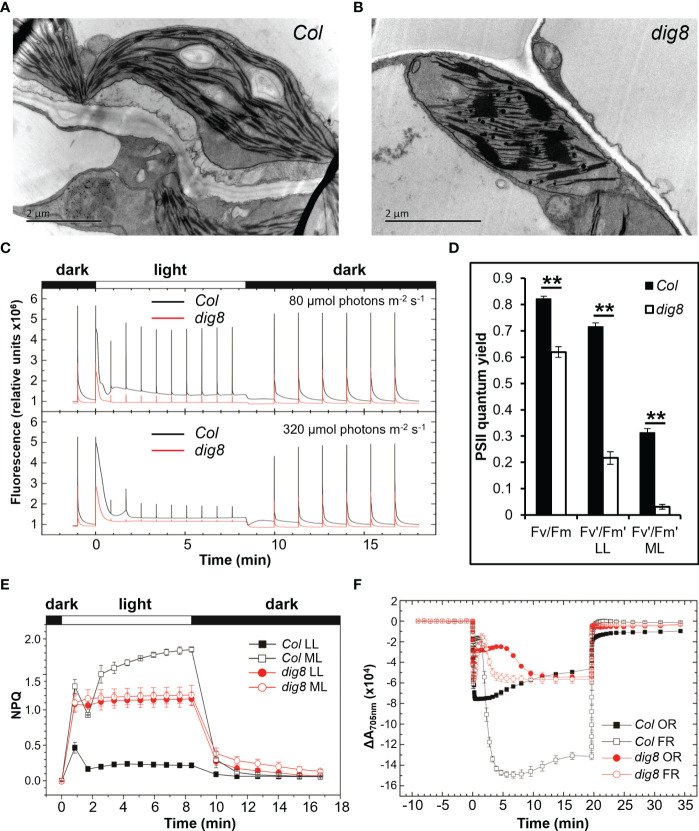
The *dig8* mutation causes aberrant grana structure in chloroplasts. A and B, images of transmission electron microscopy of grana structures in leaf chloroplasts of Col **(A)** and *dig8* mutant **(B)**. Scale bar, 2 μm. **(C)**, Fluorescence of Col and *dig8* mutant measured by a Joliot-type spectrophotometer. Leaves of Col and *dig8* mutant were dark-adapted for 15 min, illuminated with either low (80 μmol photons m^-2^ s^-1^) or medium (320 μmol photons m^-2^ s^-1^) orange actinic light (630 nm), and allowed to recover in darkness. Saturating pulses (7,900 μmol photons m^-2^ s^-1^, 200 ms duration) were applied to measure maximum fluorescence Fm and Fm’. **(D)**, maximum PSII quantum yield of Col and *dig8* mutant in darkness and light. Darkness adapted Arabidopsis leaves were exposed to different intensities of actinic light followed by recovery in darkness. Maximum quantum yield of PSII was calculated. LL: 80 μmol photons m^-2^ s^-1^, ML: 320 μmol photons m^-2^ s^-1^. **P < 0.01 (Student’s *t* test). **(E)**, Chlorophyll a fluorescence quenching of Col and *dig8* mutant. Leaves of Col and *dig8* mutant were darkness-adapted for 15 min. NPQ was induced by 500 s of orange actinic light (630 nm, LL: 80 μmol photons m^-2^ s^-1^, ML: 320 μmol photons m^-2^ s^-1^), followed by recovery in darkness. Four independent biological replicates were analyzed. **(F)**, P_700_ oxidation and reduction in Col and *dig8* mutant. Darkness adapted (15 min) leaves of Col and *dig8* mutant were illuminated with orange (630 nm, 320 μmol photons m^-2^ s^-1^) or far-red (725 nm, 1400 μmol photons m^-2^ s^-1^) actinic light. P_700_ oxidation and reduction were monitored by absorbance changes at 705 nm. Four independent biological replicates were analyzed.

Since DIG8 was localized in chloroplasts and had an essential role in chloroplast protein synthesis, mutation in the *DIG8* allele would likely cause changes in the chloroplast structure. Transmission electron microscopy of leaf cross-sections of Col and *dig8* mutant showed intact and evenly distributed thylakoid layers in Col, whereas aggregations of thylakoid that formed 4-5 giant stacks were evident in the mutant (**Figures 5A, B**). This abnormal thylakoid stacking alone would lead to chloroplast dysfunction. Although DIG8 was a component of the peptide translation process, mutation of *DIG8* would obviously affect expression of chloroplast encoded genes. Indeed, we found some of chloroplast genes were down-regulated, whereas others were up-regulated (**Figure S7**).

Since the grana in the *dig8* mutant were significantly changed, the photosynthetic properties of Col and *dig8* mutant plants were analyzed. PSII performance was monitored by chlorophyll a fluorescence. The *dig8* mutant showed a much lower chlorophyll fluorescence level than the WT (**Figure 5C**). The maximum PSII quantum yield of *dig8* mutant was about 75% of Col. The steady-state PSII quantum yield (Fv’/Fm’) of *dig8* under low light (80 μmol photons m^-2^ s^-1^) was reduced to 30% of the Col. With light intensity increased to 320 μmol photons m^-2^ s^-1^, Fv’/Fm’ of the *dig8* mutant dropped further to 13% of Col (**Figure 5D**). Under 80 μmol photons m^-2^ s^-1^ illumination, Col did not induce obvious NPQ, whereas the *dig8* had much higher NPQ. Under moderate light illumination (320 μmol photons m^-2^ s^-1^), NPQ was significantly induced in Col compared to the low light conditions, whereas the *dig8* mutant showed no change in NPQ value compared to its low light value and was significantly lower than that of the WT. After illumination NPQ was allowed to relax in darkness. The recovery of the *dig8* mutant was slower than that of Col (**Figure 5E**).

P_700_ oxidation in light followed by ${\text{P}}_{700}^{+}$ reduction in dark was analyzed to examine the function of PSI. When leaves were illuminated with saturating far-red light, only PSI was excited. The amplitude of absorbance change reflects the amount of excited PSI reaction centers. As shown in **Figure 5F**, the amount of active PSI reaction centers in the *dig8* mutant was about 45% of that in the WT, levels that agreed with the amounts of chlorophyll in the respective plants (**Figure 1D**). When Col samples were illuminated with orange light, both PSI and PSII were excited. Considerable amounts of electrons fluxed from PSII to the PQ pool, which reduced ${\text{P}}_{700}^{+}$ to P_700_, caused a decrease in the final amount of oxidized PSI reaction centers. However, the *dig8* mutant showed almost the same amount of ${\text{P}}_{700}^{+}$ when excited by orange light and far-red light, suggesting there was much less electron input from PSII in the *dig8* mutant.

### 2.5 Enhanced ROS level and callose deposition in the *dig8* mutant

Excessive light can damage relevant organelles involved in photosynthesis. Therefore, plants need NPQ for protection. Under the action of NPQ, excess light energy is lost in the form of heat. Since *dig8* mutant induced high NPQ even under low light conditions (**Figure 5E**), we speculated that low light intensity for Col was equivalent to strong light stress for the *dig8* mutant. Thus, the *dig8* mutant would exhibit stress symptoms of high light intensities even under conditions of normal light intensity.

**Figure 6:**
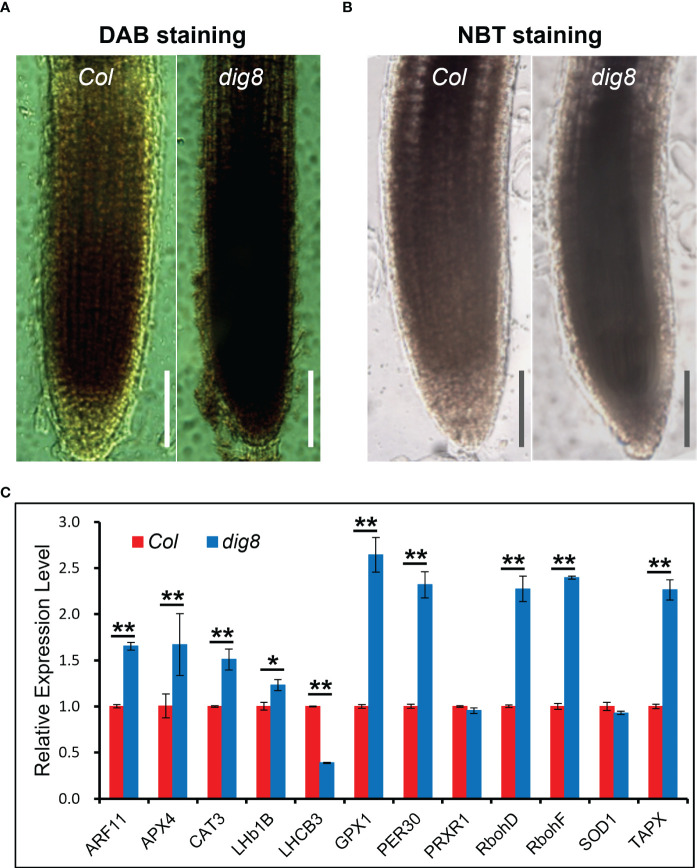
Higher ROS levels in the *dig8* mutant. A and B, ROS was detected by DAB **(A)** and NBT **(B)** staining. Seedlings of Col and *dig8* mutant were immersed in DAB and NBT solution at room temperature, and decolorization in ethanol. The images were captured with a Leica M80 stereo microscope. Scale Bar, 1 mm. **(C)**, qRT-PCR analysis of ROS-related genes in Col and *dig8* mutant. Error bars represent SDs among three independent replicates. Asterisks indicate a significant difference between the indicated samples (*t*-test, *P < 0.05, **P < 0.01).

To test this hypothesis, ROS levels were detected by DAB and NBT staining. As shown in **Figures 6A, B**, tissues of the *dig8* mutant stained much more darkly than Col, indicating that ROS generation in *dig8* mutant cells was much greater than in Col. The stained signal intensity was quantified by Image J (**Figure S8**). The ROS-related gene expression levels were mostly increased in the *dig8* mutant, consistent with higher ROS levels (**Figure 6C**).

**Figure 7:**
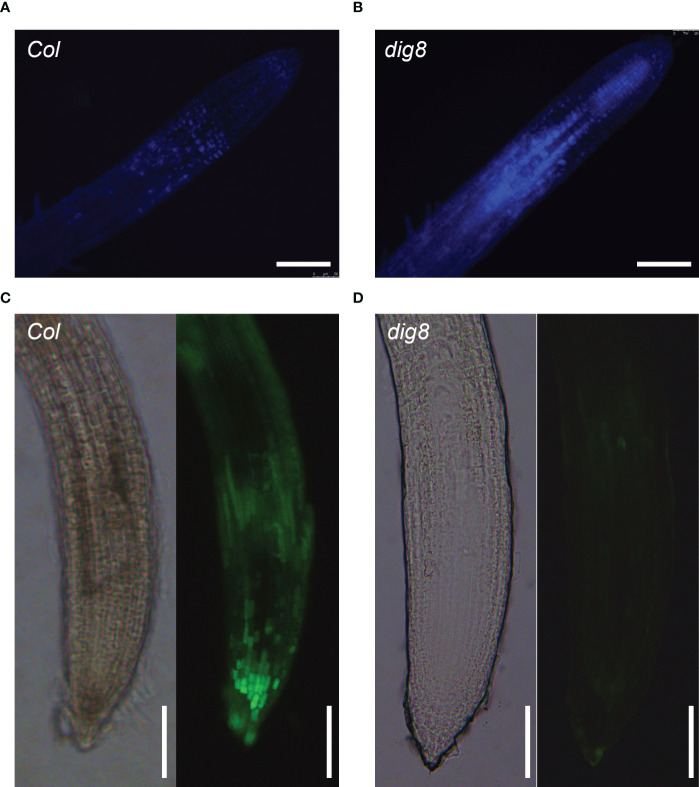
ROS caused lower PD permeability in the *dig8* mutant. **(A, B)**, Callose deposition in Col and *dig8* mutant. Seedlings were cleared and stained with aniline blue. Roots images were taken with fluorescence microscopy. **(C, D)**, PD permeability of Col and *dig8* mutant. Cotyledons of 10-day-old seedlings were wounded and CFDA dye solution was applied to the wound. Roots images of Col and *dig8* mutant **(D)** were taken with a microscope. Scale bar, 1 mm.

Aniline blue staining of roots of Col and *dig8* mutant was undertaken to test if excessive ROS levels in the *dig8* mutant caused increased callose deposition. The mutant showed much more callose deposition than Col (**Figures 7A, B**). Among the 12 *GSL* genes in *Arabidopsis*, *GSL8* was the major one responsible for callose synthesis and mutations of *GSL8* resulted in severe growth retardation. Expression levels of *GSL* genes in Col and *dig8* mutant seedlings were determined under normal growth conditions. As shown in **Figure S9**, 6 of these *GSL* genes were upregulated in the *dig8* mutant and among them, *GSL8* was expressed fourfold more that in Col. Thus excessive accumulation of callose in surrounding walls could restrict phloem unloading and cause constriction of intercellular symplastic channels. To test the PD permeability among plant cells, CFDA (5, 6-carboxyfluorescein diacetate), a fluorescent dye capable of penetrating cell membrane and plasmodesmata, was used as a probe for cell labeling. 10-d-old seedlings were wounded on cotyledon by scissors and CFDA solution was applied to the wound. The fluorescent signals were detected after incubation for 10 min allowing molecular transportation through plasmodesmata. As shown in **Figures 7C, D**, the CFDA signal could be detected at root tip of Col, but hardly any signal was observed at the whole root of *dig8.* These observations suggest that the molecular transportation through plasmodesmata from shoot to root was largely blocked in *dig8* mutant.

### 2.6 *dig8* mutant can be rescued by locally sugar supplement

**Figure 8:**
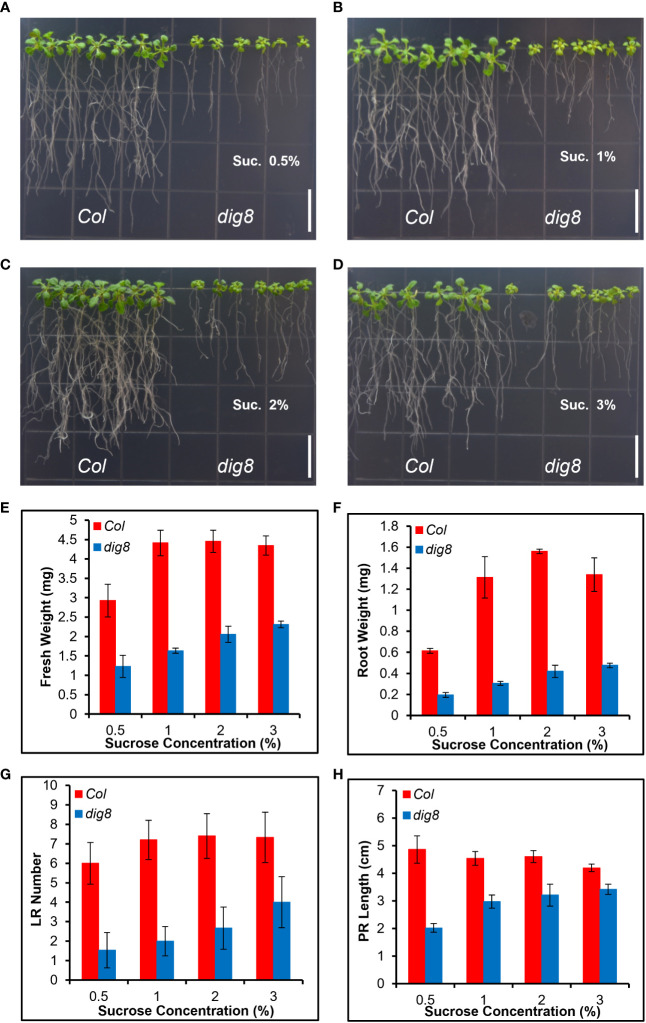
The *dig8* mutant phenotype can be partially rescued by sugar supplementation. **(A–D)**, Col and *dig8* mutant grown on MS media supplemented with sucrose at the rate (w/v) of 0.5% **(A)**, 1% **(B)**, 2% **(C)**, or 3% **(D)**, respectively. Scale bar, 1 cm.10-d-old seedlings were transferred to the shown vertical plates and pictures were taken 2 weeks after the transfer. **(E–H)**, fresh weight **(E)**, root weight **(F)**, lateral root number **(G)** and primary root length **(H)** of Col and *dig8* mutant seedlings at different sucrose concentrations. Error bars represent SD (*n*=20).

Since lower PD permeability was found in *dig8* mutant caused by increased ROS level and callose deposition, we tested whether an increased local carbon source in the growth medium could rescue the mutant phenotype. Sucrose at 0.5%, 1%, 2% and 3% (W/V) was added into the growth medium and the growth status were recorded 14d after the seedlings were transferred to the plates. As shown in **Figures 8A–D**, comparing with Col, the *dig8* mutant exhibited significant increases in primary root length, lateral root number, and biomass, particularly at higher sucrose levels. With biomass of Col values reach peak at 1% sucrose, *dig8* biomass increased 2 times without sucrose concentration limitation. The similar tendencies were found in root weight, LR number and PR length (**Figures 8E–H**). These results demonstrated that a locally nutrient supplement largely rescued the cell energy deficit.

## 3 Discussion

### 3.1 DIG8 protein is essential for plant growth

In this study we identified and characterized a mutant *dig8* that is defective in lateral root development. Map-based cloning showed that *DIG8* encodes a peptide chain release factor in chloroplasts that is essential for plant growth and development. The *DIG8* gene is identical to the *APG3* gene whose mutation caused an albinotic or pale green phenotype. While homozygous T-DNA insertion mutant *apg3*-3s (Motohashi et al., 2007; Satou et al., 2014) is not viable, the *dig8* mutation is viable albeit grew poorly. The *dig8* mutation caused a single base change in the functional PCRF domain of the protein (**Figure S4**) and had a less drastic phenotype. The more severe phenotypes seen with the F_1_ plants derived from the *dig8* and *apg3-3s* cross were likely due to the dosage effect of the single copy of the *dig8* weak allele whereas the other *apg3-3s* copy (a T-DNA insertion) was null.

In addition to ribosome release factors many plastid-located proteins involved in protein translation affect chloroplast development and plant growth with somewhat similar effects. These include chloroplast chaperonin 60 (Apuya et al., 2001; Suzuki et al., 2009; Ke et al., 2017), chloroplast translation elongation factor G (Albrecht et al., 2006; Ruppel and Hangarter, 2007), chloroplast ribosome recycling factor (Wang et al., 2010), and plastid ribosomal protein (Ma et al., 2015). Accuracy and efficiency of the chloroplast translation system is essential for chloroplast function, and termination of protein translation must occur for protein release and ribosome recycling. It seems likely that peptide chain release factor DIG8 in the chloroplast translation system has a similar function to bacterial release factors, which promote hydrolysis of peptidyl-tRNA during termination of translation (Adio et al., 2018).

### 3.2 DIG8 affects redox homeostasis and callose accumulation in plant

Chloroplast-to-nucleus retrograde signaling is essential for plant cell function. The chloroplast is a major site of reactive oxygen species (ROS) production (Koussevitzky et al., 2007). As a chloroplastic protein with important function, DIG8 affects chloroplast development and functions. Mutations of DIG8 may result in slower dissociation of nascent peptides from ribosomes in chloroplasts, and in turn reduce the translation efficiency of chloroplast-coded genes, most of which are core components of photosynthesis. Indeed, the dig8 mutation leads to abnormal thylakoid stack (**Figures 5A, B**) and extremely poor PSI and PSII performance (**Figures 5C–F**).

The link between ROS levels and callose deposition has been demonstrated in many studies. Callose biosynthesis and deposition is induced by ROS which can be triggered by dysmorphism of chloroplasts, exposure to stress conditions and pathogen attack (Dat et al., 2000; Bolwell et al., 2002; Bussotti et al., 2005; Kissoudis et al., 2016; D'Ambrosio et al., 2017). We found that, with most of callose biosynthesis gene *GSLs* highly expressed, callose is highly deposited in *dig8* mutant (**Figure S9**).

### 3.3 DIG8 regulates plant growth by affecting sugar transport and photosynthetic efficiency

As the most important transportation form of sugars, sucrose transportation is very important for plant growth and biomass accumulation. By adding sucrose in growth medium, the *dig8* mutant can be largely rescued, demonstrating that chloroplast has a strong correlation with PD-mediated sugar transportation (**Figure 8**). Similarly, a sucrose transportation reporter system pSUC2-GFP was used to screen trafficking mutants in maize. Maize *gat1* mutant was isolated and mapped to a gene encoding a plastid thioredoxin (TRX-m3) that has antioxidant function in post-translational protein redox status, indicating chloroplast affects sucrose transportation by redox-mediated PD dimension(Benitez-Alfonso et al., 2009).

In addition, DIG8 also regulates plant growth by affecting photosynthesis efficiency. As well as photosynthesis, light can damage photosynthetic apparatus. Irreversible photosynthetic apparatus inactivation produces an inhibitory effect on photosynthesis, which is called photoinhibition. To reduce photoinhibition, plants have the rapid quality control of PSII against photooxidative damage. In this repair cycle, D1 protein synthesis is the key step. Mutation of DIG8 will decrease the D1 protein dissociation efficiency from ribosomes, and in turn can repress PSII system reestablishment for photosynthesis. Actually, researchers had allotropic expressed of the psbA gene in Arabidopsis, tobacco and rice, and found that more D1 protein significantly stimulates transgenic plant growth by enhancing net CO_2_ assimilation rates with increases in biomass and grain yield (Chen et al., 2020). Overexpression of DIG8 may promote plant growth and could be exploited in crop breeding.

## 4 Conclusions

In this study, we found that the functional loss of the peptide chain releasing factor *DIG8* in *Arabidopsis thaliana* led to abnormal development and function of chloroplasts and over accumulation of ROS and callose, resulting in obstruction of redox-mediated PD transportation between plant cells which ultimately led to the abnormal development of plants. Our research shows that DIG8 is essential for plant growth.

## 5 Materials and methods

### 5.1 Plant material and growth conditions

The Arabidopsis *dig8* mutant in Col background was isolated from an EMS mutant library. The EMS mutant library was generated in our lab by ethyl methanesulphonate (EMS) mutagenesis of Col ecotype. An *apg3-3s* (SAIL_1251_H05) heterozygous mutant was ordered from ABRC (Arabidopsis Biological Resource Center, Ohio State University, Columbus OH, U.S.A.). Transgenic P_DIG8_-GUS lines were generated by floral dipping. Seeds were sterilized in 50% bleach and rinsed with water before sowing on half MS medium and incubated at 4°C for stratification. Seedlings were grown in growth rooms at 21°C with a 14 h-light/10h-darkness photoperiod.

### 5.2 Measurement of chlorophyll contents

Leaves were harvested, weighted and placed in 1.5 mL tubes; 80% acetone was added and the samples were incubated in darkness for 2 h. The samples were centrifuged and supernatants were transferred to new tubes. Light absorptions at 665 nm and 649 nm were measured with a microplate reader and were used to determine chlorophyll concentrations.

### 5.3 GUS staining

Col expressing the *DIG8* promoter (2000 bp) driven GUS (*P_DIG8_-GUS*) was used to determined expression pattern of the *DIG8* gene. For GUS-staining, plant tissues were immersed in the GUS staining buffer and incubated overnight at 37°C. After removal of the staining buffer the samples were washed with 70% ethanol and GUS signals were captured with a Leica M80 stereo microscope.

### 5.4 Protoplast isolation and transient expression analysis

Protoplast isolation and transient expression assays were performed followed standard protocols (Yoo et al., 2007). Briefly, 3-week-old young leaves were cut to 1 mm strips and the cell walls were digested by the fungal Cellulose R10^®^ and Macerozyme R10^®^. Centrifuge the digested mixture at 100g to pellet the protoplasts in a 30-ml round-bottomed tube for 1-2 min. Re-suspend protoplasts in W5 solution. Rest the protoplasts by keeping on ice for 30 min. Remove the W5 solution as much as possible without touching the protoplast pellet. Re-suspend protoplasts in MMG solution and keep at room temperature. Transient expression was performed in a 2-mL microfuge tube, 10 µL DNA were added to tube and then gently mixed with 100 µL protoplasts. After adding 110 µL of PEG, the tube was gently tapped to allow completely mixing. Incubate the transfection mixture at room temperature for up to 15 min. Dilute the transfection mixture with 400-440 µL W5 solution and mix well to stop the transfection process. Centrifuge at 100*g* for 2 min using a bench-top centrifuge and remove the supernatant. Re-suspend protoplasts gently with 1 ml WI in each well of a 6-well tissue culture plate. Incubate protoplasts at room temperature (20-25°C) for 16 h. For subcellular localization, the DIG8-EYFP construct and chloroplast-localized pt-mCherry marker were co-transformed into protoplasts. EYFP and mCherry signals were detected by using a Zesis LSM710 confocal microscope. For protein-protein interaction (BiFC) analysis, the DIG8-c-EYFP construct was co-transformed in combinations with n-EYFP constructs. EYFP signals were detected by confocal microscopy.

### 5.5 Gene quantification analysis

For gene quantification analysis, 14-d-old seedlings of Col and *dig8* lines were harvested and total RNA was extracted. After reverse transcription, the expression levels of each gene were checked by qRT-PCR. *ACT2* was used as control.

### 5.6 Yeast two-hybrid analysis

Yeast transformation was conducted as described previously (Cheng et al., 2018). *DIG8* was subcloned into pGBK-T7 and CPN60β, CPFTF, CPRRF and PSRP2 were subcloned into pGAD-T7. All construct combinations, including positive and negative controls, were co-transformed into yeast strain AH109 and selected on the DDO (SD-TL) medium. Protein interactions were displayed on X-gal DDO and TDO (SD-TLH) media. Briefly, pick one colony of Y2Hgold yeast cells in 2 ml YPD, incubate at 30°C overnight with shaking at 200 rpm. Centrifuge at 10,000 rpm for 1 min, discard supernatant. The pellet was resuspended in LiAc and brief centrifugated, and resuspended in 450 μl LiAc. After centrifugation at 10,000 rpm for 1 min and discarding supernatant, yeast cells were mixed with DNA. After adding 40 μl DMSO, the mixture was vortexed briefly and incubated at 42°C in a water bath for 20 min. The mixture was chilled on ice for 3 min and briefly centrifuged. After suspending in 100-150 µl H_2_O, cells were spread on the SD drop-out medium.

### 5.7 Transmission electron microscopy

Leaves of Col and *dig8* mutant were collected and cut into small pieces before fixation in glutaraldehyde solution followed by OsO_4_ treatment. After washing with PBS buffer, the samples were dehydrated in ethanol and embedded into Epon 812 resin. Chloroplast structures of Col (*n*=12) and *dig8* (*n*=15) in ultrathin sections were observed with a transmission electron microscope, at least 3 ultrathin sections of each were examined.

### 5.8 PSII fluorescence and oxidation-reduction measurements

PSII fluorescence quenching and ${\text{P}}_{700}^{+}$ oxidation-reduction were monitored using a Joliot spectrometer JTS-10 (Bio-Logic Scientific Instruments). Arabidopsis leaves were dark-adapted for 15 min prior to initiating measurements. Chlorophyll a fluorescence traces of leaves were recorded in fluorescence mode. Non-photochemical Quenching (NPQ) was induced by 500 s of orange actinic light (630 nm, low intensity: 80 μmol photons m^-2^ s^-1^ or moderate intensity: 320 μmol photons m^-2^ s^-1^) and plants were allowed to recover in darkness. Saturating pulses (7,900 μmol photons m^-2^ s^-1^, 200 ms duration) were applied to measure maximum fluorescence Fm and Fm’. The maximum PSII quantum yield (Fv/Fm) and steady-state PSII quantum yield in light (Fv’/Fm’) were calculated. NPQ was calculated by (Fm – Fm’)/Fm’. For P_700_ measurements, dark-adapted leaves were illuminated with orange (630 nm, 320 μmol photons m^-2^ s^-1^) or far-red (725 nm, 1400 μmol photons m^-2^ s^-1^) actinic light. The amount of ${\text{P}}_{700}^{+}$ was recorded by absorbance change at 705 nm during the actinic pulse and following darkness. All experiments were repeated with 10 leaves of each Col and *dig8* mutant.

### 5.9 ROS detection

ROS levels were detected by DAB and NBT staining. For DAB staining, seedlings of Col and *dig8* mutant were immersed in DAB solution for 24 h at room temperature. The seedlings were decolorized in 95% ethanol for 0.5 h and the remaining brown polymerization product produced by DAB interaction with ROS was observed using a Leica M80 stereomicroscope. For NBT staining, seedlings of Col and *dig8* mutant were immersed in NBT solution at room temperature for 24 h without light. The following procedures were the same as for DAB staining. For ROS signal quantification, the DAB and NBT staining images of the seedlings to be analyzed were imported to ImageJ, and converted into 8-bit grayscale images. Then adjust the threshold to select the stained part of the seedling and calculate the stained area to quantify ROS signal intensity of Col (*n*=5) and *dig8* (*n*=5) mutant.

### 5.10 Aniline blue staining and PD permeability test

Callose structures were revealed by aniline blue staining. Tissue samples destained overnight in 95% ethanol were washed in PBS buffer before immersion in callose staining buffer with 0.01% aniline blue for 2 hours and imaged in Citifluor solution under fluorescence microscope with UV filters. For PD permeability test, cotyledons of 10-day-old Col and *dig8* seedlings were wounded with scissors, and CFDA dye solution was dropped on the wound. After 10 min incubation, the samples were observed under fluorescence microscope.

## Data availability statement

The original contributions presented in the study are included in the article/**Supplementary Material**. Further inquiries can be directed to the corresponding authors.

## Author contributions

TC and LX conceived and designed the research. XZ, YH, and XH performed the experiments. TC wrote the manuscript. SZ provided technical assistant. XZ, YH, and XH contributed equally to this work. All authors contributed to the article and approved the submitted version.
