# Supplementary material for: Peptide chain release factor DIG8 regulates plant growth by affecting ROS-mediated sugar transportation in Arabidopsis thaliana
Source: Front Plant Sci. 2023 Mar 31;14:1172275. doi: 10.3389/fpls.2023.1172275 (PMC10102589; doi:10.3389/fpls.2023.1172275)
Supplement: Supplementary file 1 [file DataSheet_1.docx]

Supplementary Material

**Peptide chain release factor DIG8 regulates plant growth by affecting ROS-mediated sugar transportation in *Arabidopsis thaliana***

**Xiangxiang Zhang^1†^, Yuliang Han^1†^, Xiao Han^2†^, Siqi Zhang^1^, Liming Xiong^3*^, Tao Chen^1*^**

*** Correspondence:**

Tao Chen, Email: chentao@hznu.edu.cn; Liming Xiong, Email: lxiong@hkbu.edu.hk.


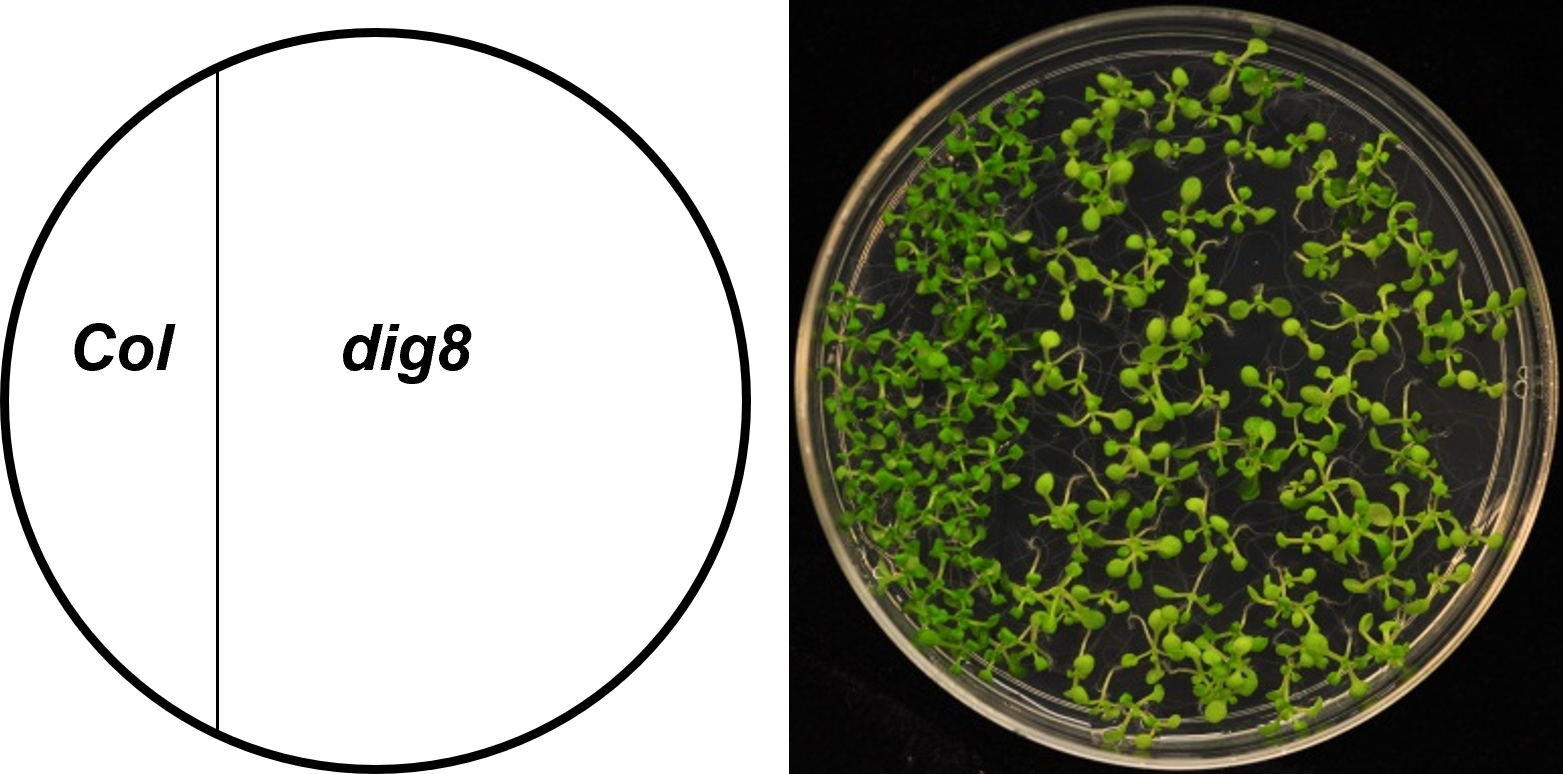


**Supplementary Figure 1.** The cotyledons and leaves of dig8 mutant are pale green. The seeds of Col and dig8 were sterilized and sown on the MS medium. The image was captured 14 days after germination.


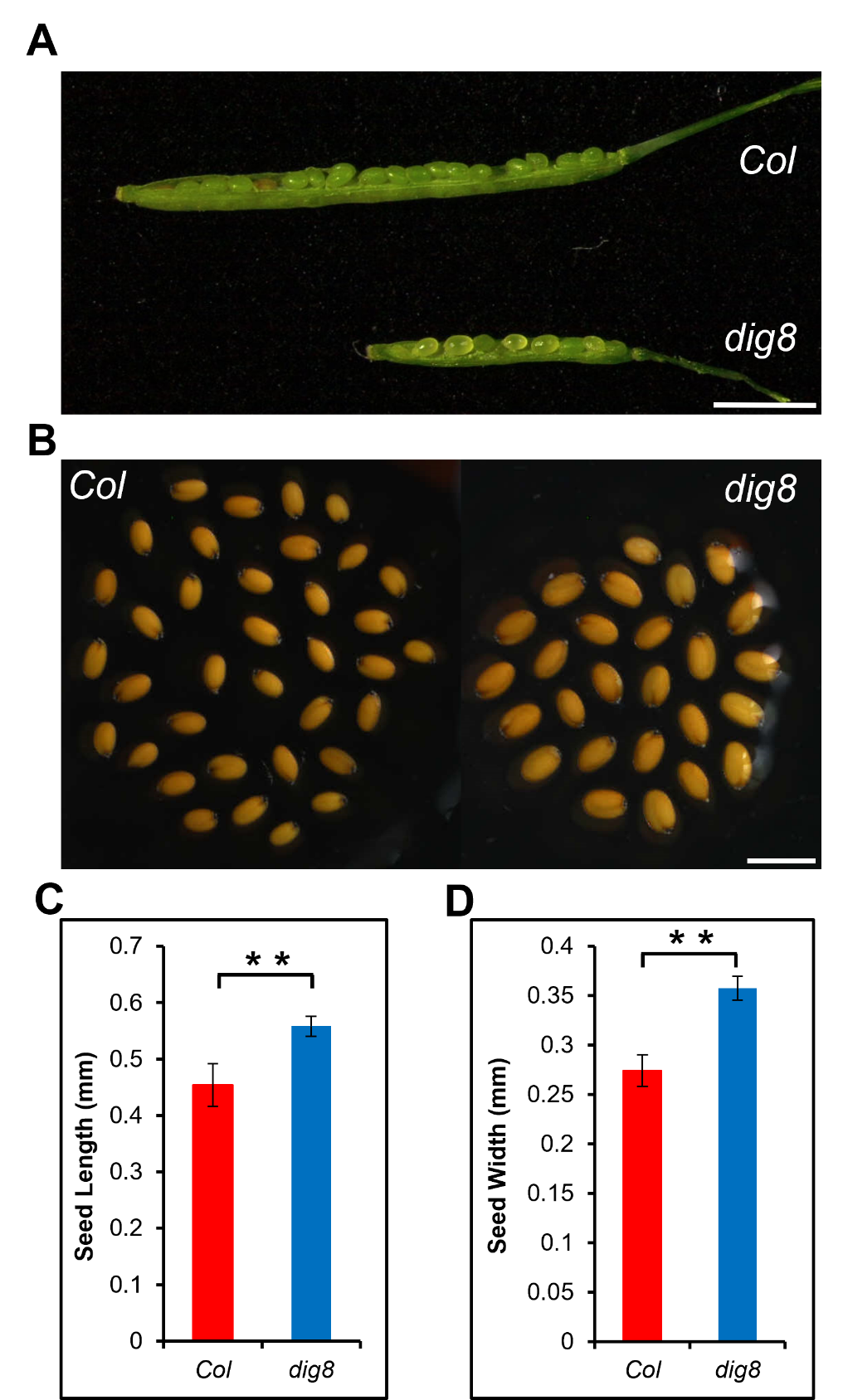


**Supplementary Figure 2.** The seed developmental phenotype of dig8. A, The siliques of Col and dig8. Scale bar, 0.25 cm. B, Matured seeds of Col and dig8. Scale bar, 1 mm. C and D, seed length (C) and seed width (D). Shown are means and SD (n=80). **P < 0.01 (Student's t test).


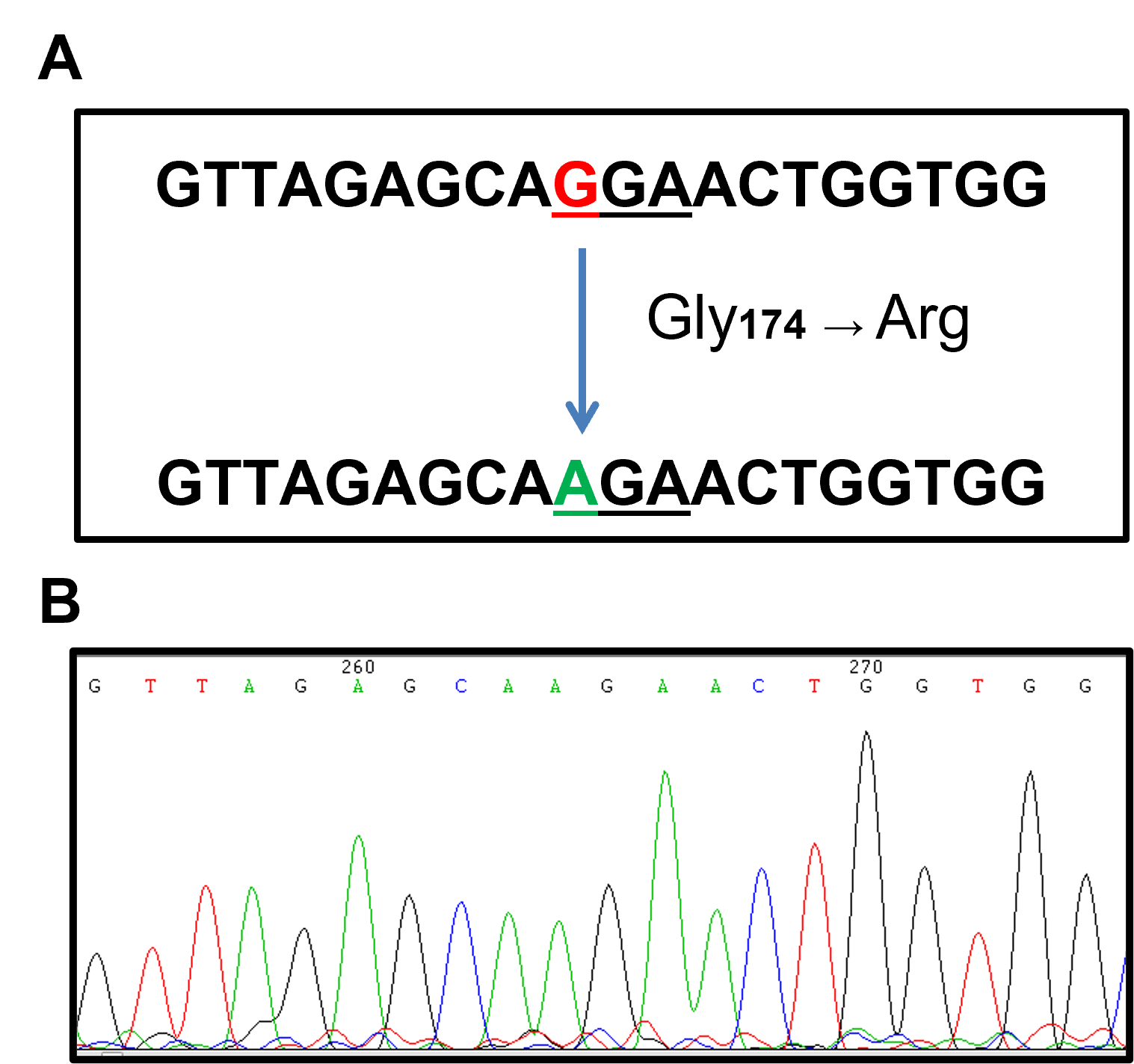


**Supplementary Figure 3.** The DNA mutation in dig8. A, The mutation of the dig8 mutant. Upper line, the sequence in Col; lower line, sequence in dig8. In dig8, G was converted to A, resulting in the change of Gly174 to Arg. B, the DNA sequence chromograph around the mutation site.


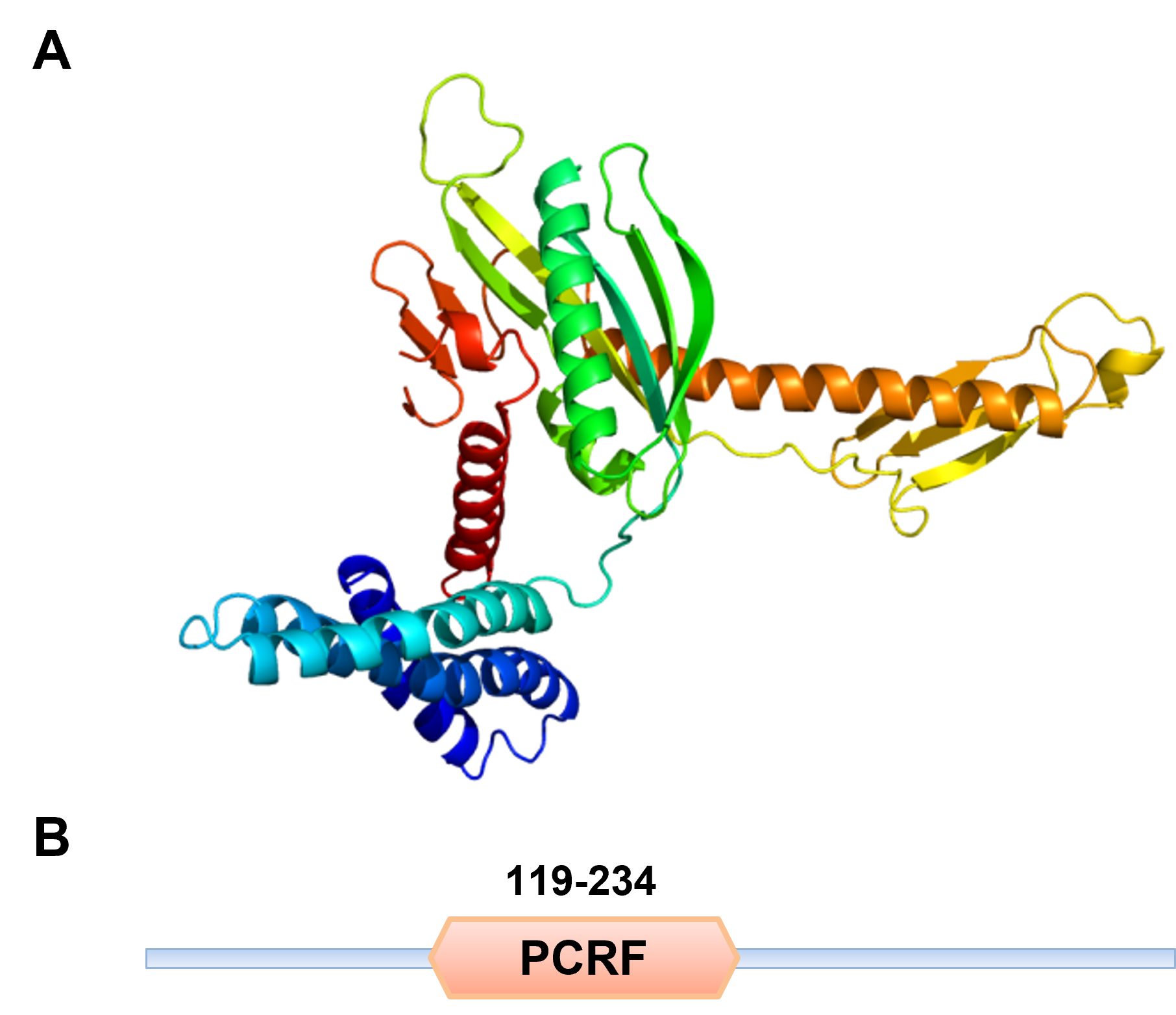


**Supplementary Figure 4.** Structure of the DIG8 protein. A, The three-dimensional structure of the DIG8 protein predicted by Phyre2. B, the domain structure of the DIG8 protein. The PCRF domain is shown.


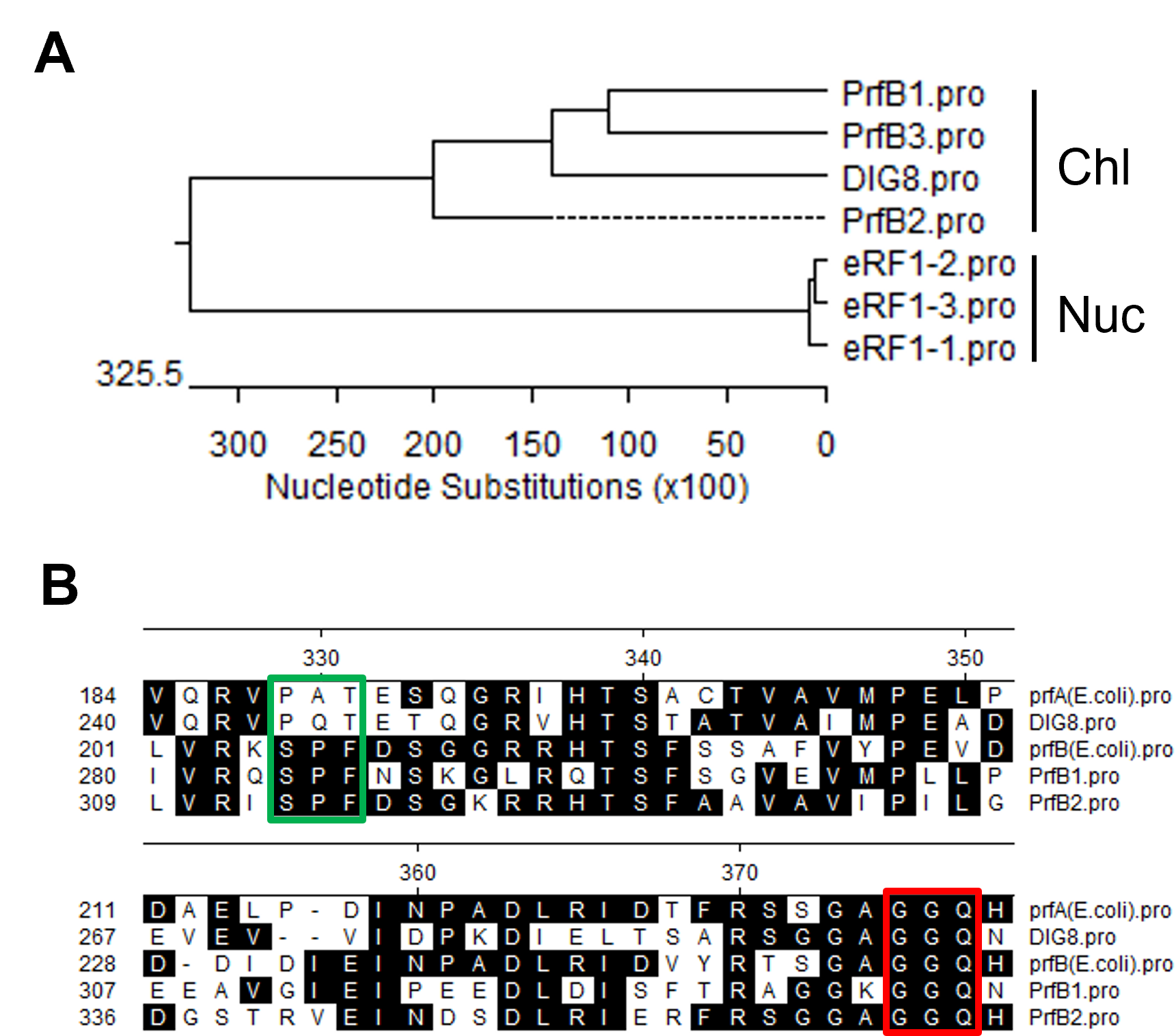


**Supplementary Figure 5.** DIG8 was predicted to be a chloroplast peptide release factor. A, Phylogenetic tree analysis of release factor in Arabidopsis. Nuc, nuclear localized; Chl, chloroplast localized. B, Functional motifs found in the protein clade. Green rectangle, stop codon recognize motif. UAA/UAG were recognized by PA(Q)T motif and UAA/UGA were recognized by SPF motif. Red rectangle, GGQ motif essential for the activity of peptidyl-tRNA hydrolysis.


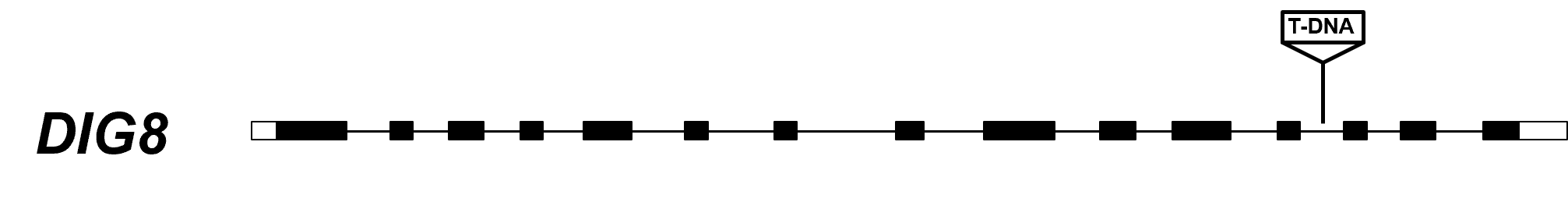


**Supplementary Figure 6.** The T-DNA insertion location of apg3-3S (SAIL_1251_H05) in the DIG8 genomic sequence. Black boxes, exons; white boxes, UTRs; lines, introns.


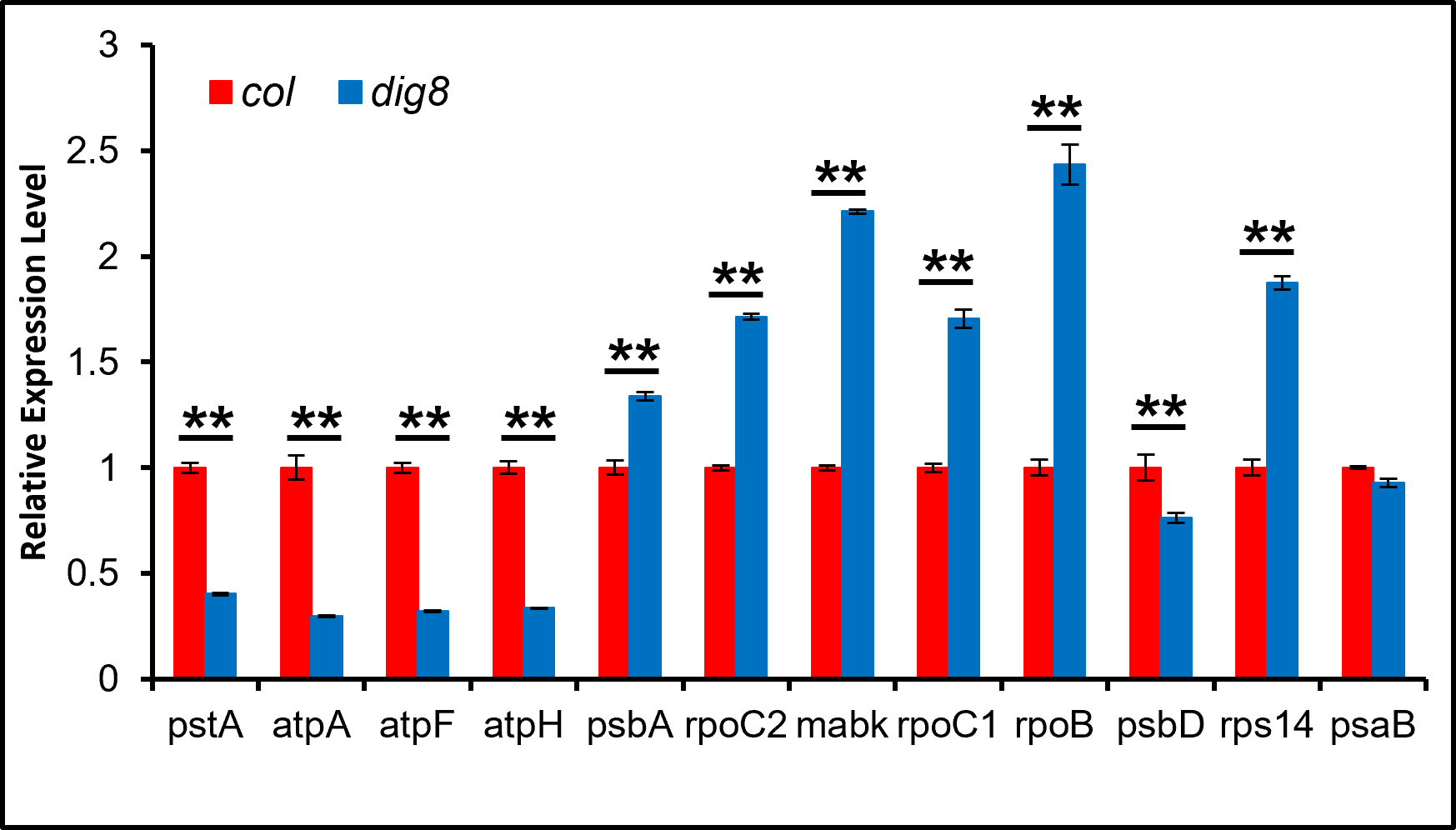


**Supplementary Figure 7.** The relative expression levels of chloroplast-code genes in Col and dig8 assessed by quantitative RT-PCR. *ACT2* was used as internal control. Error bars represent SD from three independent replicates. Asterisks indicate a significant difference between the indicated samples (Student's t test, **P < 0.01).


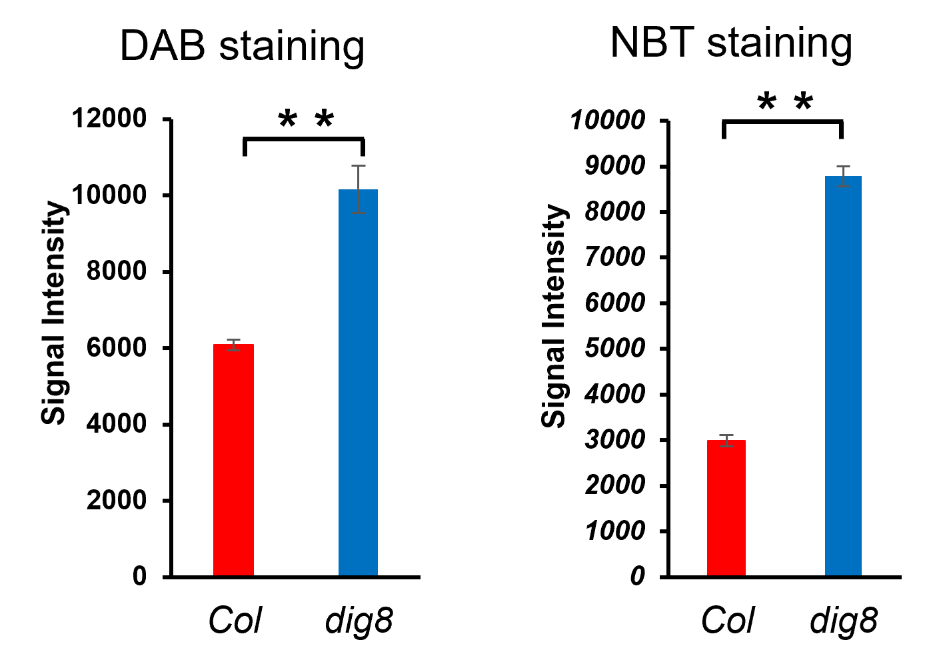


**Supplementary Figure 8.** Quantification of ROS levels in Col and dig8 seedlings. The original images of ROS in Figure 6 were quantified by Image J. **P < 0.01 (Student's t test).


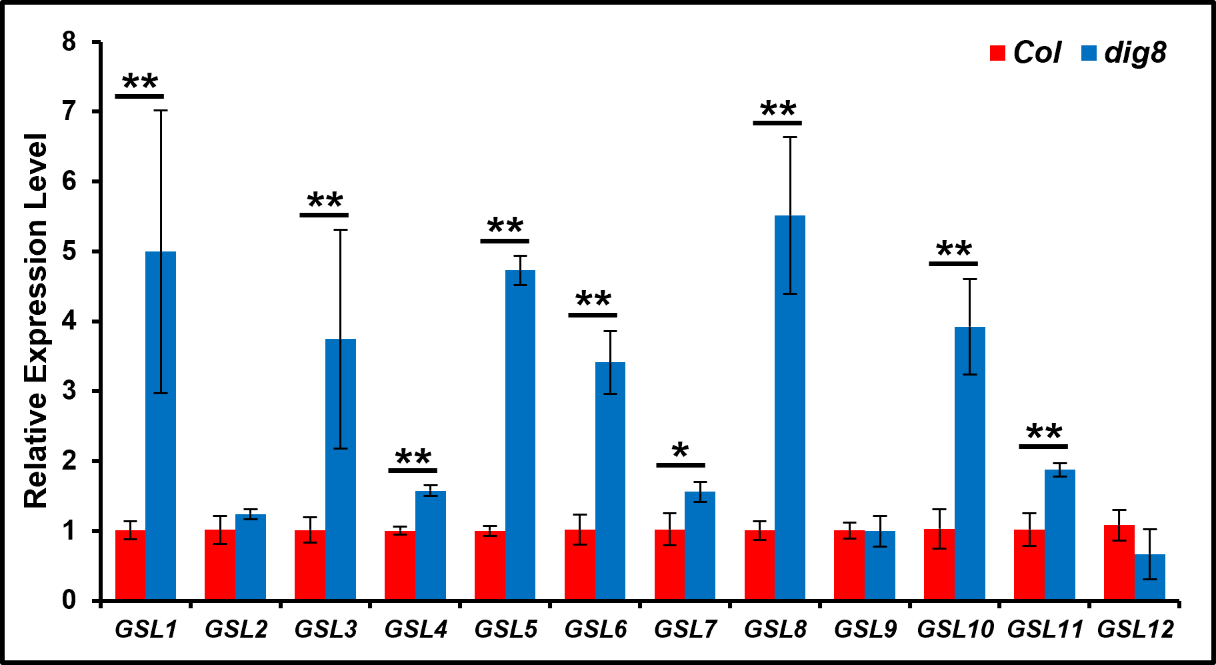


**Supplementary Figure 9.** Relative expression levels of GSL genes in Col and dig8 assessed by quantitative RT-PCR. ACT2 was used as internal control. Error bars represent SD from three independent replicates. *P < 0.05, **P < 0.01 (Student's t test).
